# Twiddler’s Syndrome Involving a Deactivated, In Situ Spinal Cord Stimulator: A Case Report

**DOI:** 10.7759/cureus.83399

**Published:** 2025-05-03

**Authors:** Kebereab Feyissa, Mark D Unger

**Affiliations:** 1 Department of Osteopathic Manipulative Medicine and Osteopathic Principles and Practices, Liberty University College of Osteopathic Medicine, Lynchburg, USA

**Keywords:** chronic pain management, interventional pain medicine, osteopathic manipulative medicine, physical medicine and rehabilitation, spinal cord stimulator, twiddler's syndrome

## Abstract

We present the case of a 63-year-old male presenting with unremitting acute flank pain ipsilateral to an implantable pulse generator (IPG) of an *in situ* spinal cord stimulator (SCS) that was permanently deactivated 12 years prior after a successful lumbar spine surgery for low back pain radiating into the bilateral lower extremities. Comparison of a current-day chest computed tomography (CT) scout film with a chest CT scout film obtained 12 years prior demonstrated rotational migration of the IPG in the patient’s flank and elongation of the extension cable. The inactive epidural lead did not show evidence of migration. Removal of the entire SCS device resulted in the resolution of the patient’s symptoms. This case stresses the importance of considering hardware-related complications when assessing new pain localized near an implant. A high level of clinical suspicion and a multidisciplinary approach were critical to the prompt management of this case.

## Introduction

Spinal cord stimulator (SCS) is indicated for the treatment of refractory chronic pain due to failed back surgery syndrome, complex regional pain syndrome, and other causes [1]. While SCS has been shown to relieve pain and improve quality of life for patients who fail conventional treatment modalities, one risk associated with SCS implantation is hardware-related complications [2]. An SCS system is composed of an implantable pulse generator (IPG), extension cables, and epidural leads [2]. The IPG is a battery-powered device that generates and controls the electrical impulses [2]. These impulses are transmitted through the extension cables to the epidural leads that deliver the electrical stimulation to the appropriate spinal level to modulate the transmission of pain signals [2].

Twiddler’s syndrome refers to the malfunctioning of an indwelling medical device due to unconscious manipulation or passive migration of one of its components [3]. In the case of SCS, flipping of the IPG and twisting of the extension cable causes migration of the epidural lead away from the targeted spinal cord region, resulting in a loss of therapeutic effect [3,4]. That scenario represents the typical presentation of Twiddler’s syndrome involving an SCS in its active state [3]. In contrast, we present a case of Twiddler’s syndrome in a patient complaining of acute flank pain 12 years after permanent deactivation of an SCS device subsequently left* in situ*.

## Case presentation

A 63-year-old male with a past medical history of hypertension, hyperlipidemia, gout, and class II obesity presented to his local emergency department with a several-hour history of severe lower back pain and spasms after bending over. At that time, magnetic resonance imaging revealed a left-sided L4-L5 paracentral disk bulge with mild-to-moderate left-sided lateral recess compression. Surgery was not pursued, and he was conservatively managed with anti-inflammatory medication and physical therapy. Over the course of two years, his symptoms progressively worsened, and he began to suffer from burning paresthesia of the bilateral lower extremities. The patient received radiofrequency ablation of the left L5 medial branch but realized temporary symptom relief. Eventually, the patient underwent an SCS trial for treatment of his chronic pain, opting for permanent implantation. The SCS leads were placed in the epidural space posterior to the T7 vertebral level, and the IPG was anchored in the subcutaneous tissue of the right flank. Initially, the patient reported favorable modulation of his bilateral burning lower extremity pain. However, eight months after implantation, the patient’s symptoms recurred. Surgical consultation identified 1+/4 patellar and ankle-deep tendon reflexes on the right with a positive straight leg raise test on the right. Computed tomography (CT) myelogram showed moderate-to-severe right-sided neural foraminal stenosis and mild left-sided neural foraminal stenosis at the L5 and S1 spinal levels. The patient elected to undergo L5 decompressive hemilaminectomy with medial facetectomy. At that time, the SCS was remotely deactivated and left *in situ* after the surgery. At follow-up several weeks later, the patient reported resolution of his back and lower extremity pain and had returned to regular work duties. Removal of the SCS was not recommended, and, given a resolution of his symptoms at that time, the SCS remained deactivated.

Twelve years after SCS deactivation, the patient presented to his primary care physician with a new complaint of sharp right-sided flank pain constant for two weeks. He denied low back or leg pain. Initial workup, including ultrasound and CT chest and CT abdomen, was unrevealing. The primary care physician referred the patient to osteopathic neuromusculoskeletal medicine (ONMM) for further evaluation and management.

**Figure 1:**
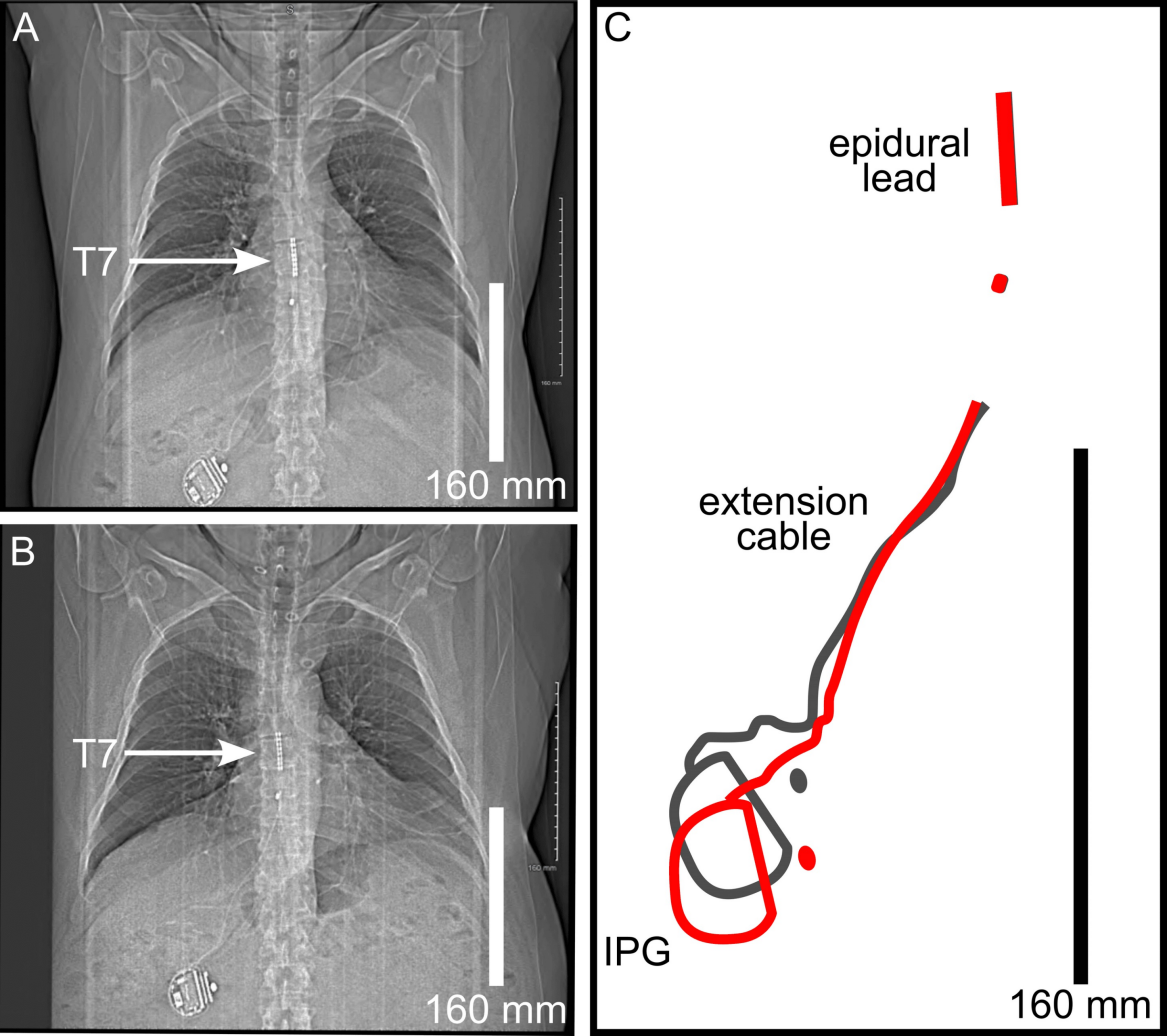
Migration of the implantable pulse generator (IPG) and extension cable. (A) Postoperative computed tomography (CT) chest scout film obtained 12 years prior to the presenting complaint of acute right-sided flank pain. (B) Current day CT chest scout film obtained 12 years after deactivation of the spinal cord stimulator (A) demonstrates rotational migration of the IPG within the patient’s right flank and elongation of the extension cable. In contrast, the epidural lead did not demonstrate migration over this 12-year period, remaining posterior to the T7 vertebral level (arrows). (C) Stylized diagram depicts rotational migration of the IPG and elongation of the extension cable (3x magnification relative to A, B). The magnitude of migration is visualized by superimposing the tracings of the IPG, extension cable, and epidural lead 12 years prior (gray lines) to the patient’s current-day (red lines) presentation in the ONMM clinic. Scale bars: 160 mm.

Physical exam by the consulting ONMM physician revealed exacerbation of the patient’s pain with gentle inferior translation of the right flank tissue and attenuation of pain with gentle superior translation. There were no discrete areas of tenderness elicited in the surrounding body wall and spine. Comparison of the most recent CT chest scout film obtained before the initial ONMM encounter with a CT chest scout film obtained after this last surgery, 12 years prior, demonstrated rotational migration of the IPG, elongation of the extension cable, and stable positioning of the epidural lead (Figures 1A-1C).

Subsequently, ONMM referred the patient to neurosurgery for consideration of SCS removal. The patient elected for removal. At the time of SCS removal by neurosurgery, all components of the device appeared to be intact without gross malfunction. Postoperatively, the patient recovered well without recurrence of his remote low back pain or lower extremity pain. Upon return to the ONMM clinic, the patient noted complete resolution of his right-sided flank pain and was recommended to return to the clinic as needed.

## Discussion

Twiddler’s syndrome was first described in association with cardiac pacemakers [5] and has since been reported in the context of other implantable devices, including SCS [3]. This condition commonly occurs due to unconscious manipulation of the SCS by the patient, which may lead to displacement of the epidural lead, hardware failure, and loss of pain relief [3]. Our current findings did not align with the existing literature, which notes that lead migration due to this condition often results in therapeutic failure and necessitates surgical intervention [3,4,6]. In our case, migration of the IPG component of a deactivated, *in situ* SCS resulted in localized flank pain ipsilateral to the IPG. To our knowledge, this finding has not been reported in the literature [3]. Of note, the SCS had been permanently deactivated 12 years after the patient’s final lumbar spine operation.

This case highlights the significance of identifying and managing complications associated with SCS and the importance of preventative strategies to mitigate Twiddler’s syndrome [4,6,7]. Additional strategies include the use of ergonomic IPG designs that use secure implantation techniques to minimize device movement [6,7], implanting the IPG in the lumbar region subcutaneously to reduce accessibility for manipulation [3], considering psychiatric interventions for at-risk SCS candidates with a history of device manipulation, cognitive impairment, or psychiatric disorders [3,8], use of the smallest possible surgical pocket, and suturing the IPG to fascia [9].

The Neurostimulation Appropriateness Consensus Committee (NACC), which includes experts in the fields of pain management, neurology, neurosurgery, and anesthesia, published comprehensive guidelines for optimization of SCS over the long term [4]. NACC guidelines note that the leading causes for loss of SCS effectiveness are lead migration or device fracture [4]. The guidelines also highlight the importance of patient selection to mitigate Twiddler’s syndrome related to SCS [3,4]. Some of these recommendations include the presence of chronic pain refractory to conservative therapies, a lack of psychiatric or cognitive impairment, and an emphasis on patient education to prevent device manipulation [4].

## Conclusions

This case emphasizes the importance of including hardware-related complications in the differential diagnosis for somatic pain near an implant site. A high level of clinical suspicion is indicated, and review of historical imaging, when available, plays an important role. This case underscores the significance of thorough assessment and multidisciplinary collaboration in the management of patients with complex pain histories.
